# How to Build a Standardized Country-Specific Environmental Food Database for Nutritional Epidemiology Studies

**DOI:** 10.1371/journal.pone.0150617

**Published:** 2016-04-07

**Authors:** Gwenola Bertoluci, Gabriel Masset, Catherine Gomy, Julien Mottet, Nicole Darmon

**Affiliations:** 1 Laboratoire Génie Industriel, CentraleSupélec, Grande Voie des Vignes, 92290, Châtenay-Malabry, France; 2 AgroParisTech, SESG UFR MIDEAL, Massy, France; 3 Unité Mixte de Recherche (UMR) “Nutrition, Obesity and Risk of Thrombosis,” Institut National de la Recherche Agronomique 1260 INRA, Institut National de la Santé et de la Recherche Médicale 1062 INSERM, Aix- Marseille Université, 13385, Marseille, France; 4 BIO by Deloitte, 92200, Neuilly-sur-Seine, France; 5 UMR GENIAL, AgroParisTech, INRA1145, Cnam, Massy, France; College of Agricultural Sciences, UNITED STATES

## Abstract

There is a lack of standardized country-specific environmental data to combine with nutritional and dietary data for assessing the environmental impact of individual diets in epidemiology surveys, which are consequently reliant on environmental food datasets based on values retrieved from a heterogeneous literature. The aim of this study was to compare and assess the relative strengths and limits of a database of food greenhouse gas emissions (GHGE) values estimated with a hybrid method combining input/output and LCA approaches, with a dataset of GHGE values retrieved from the literature. France is the geographical perimeter considered in this study, but the methodology could be applied to other countries. The GHGE of 402 foodstuffs, representative of French diet, were estimated using the hybrid method. In parallel, the GHGE of individual foods were collected from existing literature. Median per-food-category GHGE values from the hybrid method and the reviewed literature were found to correlate strongly (Spearman correlation was 0.83), showing similar rankings of food categories. Median values were significantly different for only 5 (out of 29) food categories, including the ruminant meats category for which the hybrid method gave lower estimates than those from existing literature. Analysis also revealed that literature values came from heterogeneous studies that were not always sourced and that were conducted under different LCA modeling hypotheses. In contrast, the hybrid method helps build reliably-sourced, representative national standards for product-based datasets. We anticipate this hybrid method to be a starting point for better environmental impact assessments of diets.

## Introduction

### Evaluating food sustainability in a public policy perspective

The Food and Agriculture Organization defines sustainable diets as “those diets with low environmental impacts which contribute to food and nutrition security and to healthy life for present and future generations. Sustainable diets are protective and respectful of biodiversity and ecosystems, culturally acceptable, accessible, economically fair and affordable; nutritionally adequate, safe and healthy; while optimizing natural and human resource” [1]. The big challenge with implementing relevant sustainable food policies is to integrate all these sustainability issues while also accounting for the broad diversity of food systems, stakeholders, and consumer dietary behaviors [2, 3]. Methods to evaluate and integrate the different sustainability dimensions are being developed but need to be more adaptable, coherent, systemic, and scalable. Collecting good quality data on all these dimensions and making it equally integratable into the common databases and models is a pressing priority for the sustainable diet agenda [4].

Relying on country-specific standardized environmental food databases may guarantee more robust estimates of the environmental impacts of diets for studies assessing the sustainability of consumer dietary behaviors. Nutritional epidemiology provides a useful and relevant framework here because it can successfully combine different food and diet dimensions in order to connect dietary intakes to health outcomes [5, 6]. Food composition databases, describing the energy and nutrient content of food items, are key instruments in nutritional epidemiology [7]. Energy and nutritional intakes are estimated based on a matrix calculation linking the quantity of each food with a food database computing the energy and nutrients provided by each ingested food. In reality, the exact nutritional composition of each food consumed is unknown, and so has to be replaced by the composition of standard food items recorded in the “food composition database” [8]. The upshot is a simplification of reality: for example, it is assumed that all oranges consumed by dietary survey participants have the same vitamin C content, i.e. the standard content, yet this is not perfectly true. Similarly, the study of the link between economic and nutritional characteristics is greatly simplified by the assumption that food is purchased at a standard price [9]. No representative survey to date has informed on both individual food consumption and the prices actually paid by each participant for their own personal foods. The development of average (or standard) price databases has now made it possible to estimate the cost of a balanced diet and to assess the impact of budget constraints on food choices and nutritional quality of food [10]. Today, both France and the US have national food price databases that are available for research purposes: the food prices are incorporated as a variable in the nutritional composition database and are treated in the subsequent analyses as an additional “nutrient” [10].

There is an urgent need to integrate standardized environmental data on food items into these food composition databases [10, 11]. Epidemiology studies analyzing the sustainability of individual diets are generally based on environmental datasets compiled or constructed from published data [12–19]. In particular, GHGE data are often based on the pioneering study of Audsley et al [20] who collated a set of UK-specific GHG emissions for a wide range of foods from the literature [12, 15, 17, 19]. However, combining data from heterogeneous studies led under different Life Cycle Assessment (LCA) modeling hypotheses or models specific to a given geographic setting or production mode can compromise the representativeness and relevance of the dataset. Data on the environmental impact of foods is sparse. Two sorts of databases are typically used: public or commercial databases (such as Ecoinvent or LCA-Food databases) or specific data coming from scientific literature and usually limited to only a few foods or agricultural commodities produced under specifically measured conditions [21–23]. Non-experts in LCA may find it difficult if not impossible to assess the relevance of environmental data sourced from commercial databases or scientific publications. Another major issue for epidemiology surveys is food representativeness. The food and food groups considered for building standardized environmental food databases have to be representative of actual national consumption and national food production modes. For instance, French consumers tend to buy UHT milk whereas in Scandinavian and the UK consumers buy fresh.

The aim of this study was to compare and assess the relative strengths and limits of two greenhouse gas emissions (GHGE) databases of foods widely consumed in France. One database was built with GHGE values gathered from the existing literature, while the other was obtained using a dedicated method combining input/output and LCA approaches, referred to below as “the hybrid method”, to estimate the GHGE values of foods representative of the current French market.

## Material and Methods

### Identification of food categories typically found in the French diet

The food items most representative of French diet were identified based on the most recent national food consumption survey. This food database was derived from the 7-day food records of a nationally representative random sample of adults (n = 2,624; age > 18 years) participating in the INCA2 cross-sectional dietary survey “*Enquête Individuelle et Nationale sur les Consommations Alimentaires*”. All the foods declared as consumed by the participants during the survey (n = 1,314 foods and beverages, including water) were listed in a survey-associated food database where foods were combined into 10 main food groups and 37 food families, classified on the basis of similar origins and shared nutritional profiles (e.g. fruits, vegetables, vegetable fats, animal fats, etc.). Within each food family, foods with the highest percentage of consumers were selected as representative of the food family, yielding a list of 391 widely-consumed foods. Foods rarely consumed by the INCA2 participants but with potential nutritional and/or environmental benefit (e.g. soy-based milk and desserts, walnuts) were also selected. This process resulted in 402 foods from the 1,314 items initially listed in the food database. These 402 foods covered 71% of the total weight intake and 66% of the total energy intake of the INCA2 study population. Here, we grouped these foods into 29 food “categories” to chart the results of this study.

### Estimation of the GHGE of individual foods using the hybrid method

Life Cycle Assessment (LCA) is widely recognized as the reference method for estimating the environmental impact of food products. Its generic methodology, and application cases, are defined by international standard ISO 14040 [24]. As defined in ISO 14040, the first step in LCA is to define the objectives of the study (eco-design of a new product, communication marketing and comparison with similar products). Key elements that need to be specified to complete this step include the perimeter of the model, the functional unit considered, and the size and nature of the cut-off applied, among others. However, an in-depth LCA of just one existing product costs too much time and money to be compatible with the magnitude of an epidemiology study. A more suitable alternative is to simplify and rationalize the means of evaluation of environmental impacts while still producing reliable data covering a wide range of products, specific markets and periods of time.

Top-down approaches like input-output (IO) offer a partial response to this problem. Based on Leontief’s proposition [25], the IO method makes it possible to linkage certain industrial activities with their consumption and production flows. In contrast to bottom-up approaches (as in the LCA method), there is no need, to make cut-offs to define which processes should be included or not. Lenzen [26] highlighted the fact that in LCAs, impact assessment errors due to truncation (in energetic systems) can reach to 50% of the assessed impact. However, the top-down approach is often used to avoid having to extrapolate the environmental impacts of very specific products to whole product groupings. A number of studies have suggested hybridizing data from the IO matrices with the LCA methodology [27–30] to improve the quality of environmental assessments of industrial sectors and product categories or to account for regional specificities. The IO method offers a framework for allocating the environmental impacts caused by industrial product categories for a specific region [31], thus meeting the needs of national nutritional surveys. In addition, national commercial data on imports and exports can serve to account for food products’ geographical origins as well as their transport impacts. Using these types of data makes it possible to define a realistic model of the national market in terms of geographical origins of food products consumed a country.

ISO 14001 specifies [32]—and the European Community recommends [33, 34]—using multi-criteria method to assess the environmental impact of food and diets. Water footprint, water pollution, GHGE impacts, and biodiversity effects are considered particularly relevant [35]. However, most studies on sustainable diets have remained heavily focused on GHGE [12–17, 19, 36–41]. Here we use the GHGE criterion as an example of the challenges involved in environmental impact assessments of food products.

**Fig 1:**
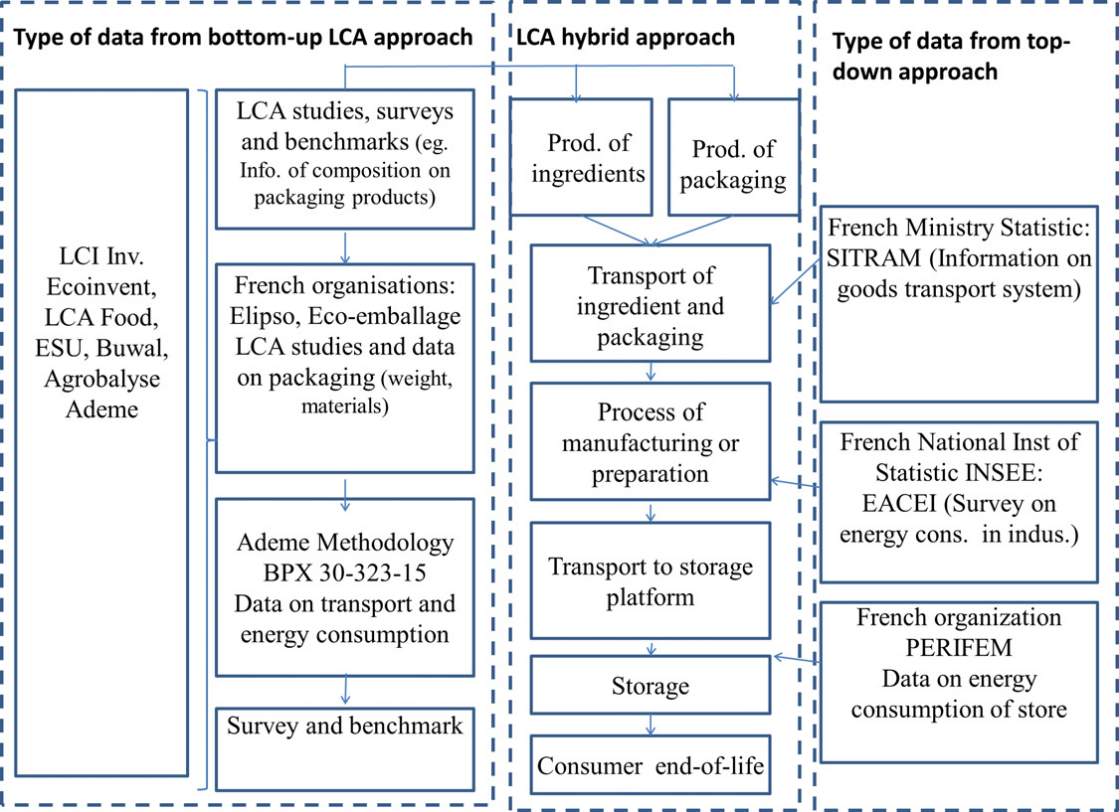
The hybrid method and knowledge database developed by Greenext

The hybrid method employed in this study is an environmental assessment method originally developed by a consultancy called Greenext to facilitate the calculation of environmental impacts for a large amount of food products. The method currently allows the assessment of GHGE (an impact measured in kg equivalent carbon. The main gases associated with the food sector are carbon dioxide and methane), eutrophication (an impact measured in kg equivalent phosphorus. The main contributors to this impact are nitrogen and phosphorus) and acidification (an impact measured in kg equivalent sulfur dioxide. The main contributors to this impact are nitrogen and sulfur compounds from energy production facilities and transportation systems). The first developments were implemented in 2008, and operationally adoption for research projects and companies took off in 2010. The Greenext methodology is based (Fig 1) on LCA standards (ISO 14040 and ISO 14044 methods) and French food products category rules [35]. The data used for each step of product life cycle is a mix of data derived from a bottom-up LCA method and a top-down IO approach. Greenext has used this hybrid method to create a knowledge database containing 1000 pre-established LCA types of food products (including different types of packaging, called “generic products”. All generic data can be replaced by specific data (e.g. allocation of co- and sub-products, subcomponents of packagings, etc.). These data have been adapted to match the national perimeter’s production and consumption characteristics. The adaptation was devised with input from expert knowledge, macroeconomic data, and distributor panels, resulting in a process that can quickly create large, specific and standardized databases of environmental impact. The study reported here used two datasets. The first set is made up of “specific data” for product categories (e.g. a cooked 15%-fat beef burger, life cycle inventories, conversion rates, packaging weights, etc.). The second set is made up of “generic” data from our study of French macro-socio-economic data (e.g. energy mix, average energy consumption of a cooler).

### Collection of GHGE levels for individual foods from existing literature

In order to compare GHGE values estimated using the hybrid method against values published in the scientific literature, we screened the literature to identify publications whose objective was to estimate the GHGE associated with individual foods or more generic food groupings. The search through existing literature was limited to the GHGE indicator as is the most common environmental impact indicator in use today. The search was conducted on March 10, 2014 targeting the following terms in the title, abstract, or as keywords: “food” and (“life cycle analys*s” or “life cycle assessment” or “LCA”) and (“carbon” or “greenhouse gas” or “GHGE” or “global warming potential” or “GWP”). Only studies dating back to the first ISO 1404X standards were retained, i.e. from 1997 onwards. The initial search identified 128, 391, 31, and 16 publications in the *Science Direct*, *Web of Science*, *Pubmed* and *Google Scholar* databases, respectively (title only was used for *Google Scholar*). A first selection of the identified publications, based on the titles and abstracts, led to the identification of 133 unique references. As a second step, we selected publications that:

- presented GHGE LCA estimates for at least one type of food,
- in which the system boundaries of the LCA were clearly defined,
- including at least one “cradle-to-farm gate” study.

For meat products, studies estimating GHGE per kg of liveweight were excluded. LCA results from 53 reviewed publications were retained using this selection process. To complete the reference list, we included publications and grey literature not identified by the existing literature search. In total, the data used came from 70 publications (S1 List).

Individual GHGE values collected from the existing literature were gathered into a dataset applying a common functional unit of 1 kg of product for comparison with the hybrid method calculation. In all the studies retrieved, the LCA system boundary was in the form of “cradle-to [exit stage]”. As a result, we identified the system boundary exit stage for each value found in the existing literature as either:

- farm gate or process plant (i.e. GHGE associated with the foodstuff’s ingredients),
- process and/or packaging (i.e. final food product before delivery to retail centers),
- transport to retail centers, retail (i.e. including emissions arising from retail steps),
- use of the product (i.e. the complete life cycle of the food product).

By searching through the existing literature, GHGE LCA estimates were obtained for 606 food items. Almost half of these values were limited to the “cradle-to-farm gate/process plant” system boundary. Only 65 values included the complete life cycle of the food items, i.e. the same system boundary as that applied in the hybrid method, and 63 values included the same scenario minus the last step of use. As the hybrid method assessed the full life cycle of the products and their usage phase accounted for less than 10% of the foods’ GHGE (in references from existing literature when the full life cycle was considered), the final comparison was conducted on these 128 (i.e. n = 65+63) values (found in the existing literature), which for 29 food categories were combined with the values calculated in these same categories using the hybrid method.

### Statistical analysis to compare literature data estimates and hybrid method estimates

Median values were calculated for each food category and for the two GHGE databases, i.e. the values obtained using the hybrid method and the values from the literature search. To assess how food categories were classified according to the hybrid method and the existing literature, Spearman rank correlations were computed on the food category medians in the two GHGE databases. In addition, the differences between the median values of each food category between the two databases were analyzed using Kruskal-Wallis tests.

Where discrepancies were found between GHGE values obtained using the hybrid method and values retrieved from the literature, additional investigations were conducted to help understand the reasons for the observed differences.

## Results and Discussion

### Hybrid method *vs* literature GHGE Estimates

**Fig 2:**
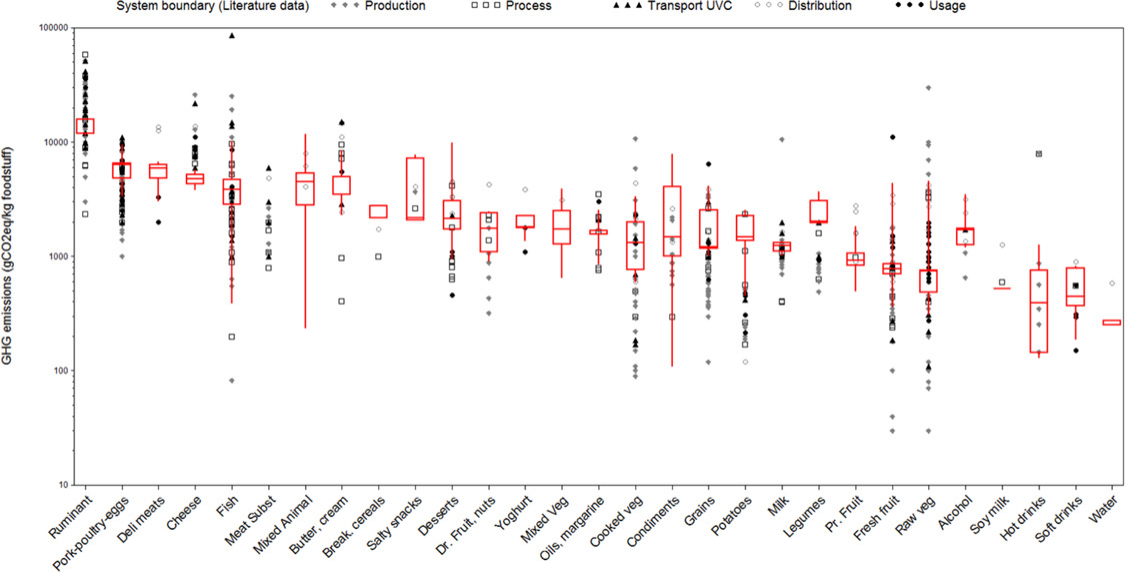
Estimates calculated with the hybrid method (Red Boxes) and retrieved from the existing literature (icons), by food category. Shape of icon indicates the system boundary considered in the publication.

**Fig 3:**
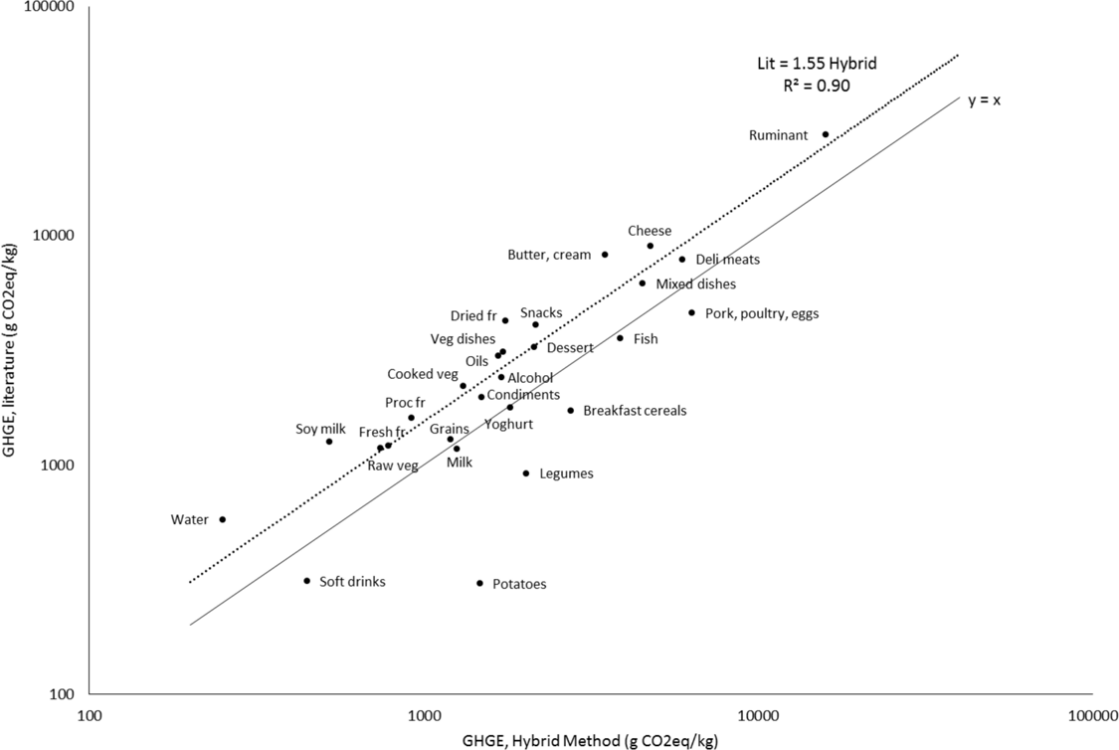
Food categories compared in terms of GHGE values from existing literature and GHGE values estimated using the hybrid method. The dotted line plots a fitted least squares regression without intercept. The solid line is the Y = X function. Dried fr, dried fruits and nuts; Proc fr, processed fruits and juices; Fresh fr, fresh fruits; Cooked veg, cooked vegetables; Raw veg, raw vegetables; Veg dishes, vegetarian mixed dishes.

Overall, the existing literature values matched well to the hybrid method estimates, as illustrated in Figs 2 & 3. The Spearman correlation of 0.83 indicates that food categories were similarly ranked with the two approaches, i.e. GHGEs estimated with the hybrid method and the values retrieved from the literature.

As shown in Fig 3, the hybrid gave lower estimates than existing literature values for all the food categories except for potatoes. Most food categories had high variability in GHGE values both in the existing scientific literature and with the hybrid method (See S1 Fig). Significant differences between existing literature and hybrid method estimates were only found for 5 (out of the 29) food categories, i.e. ruminant meats, cheeses, potatoes, raw vegetables, and fresh and processed fruit (Fig 3).

### Possible origins of variability in GHGE estimates–beef as case study

There are two sources of potential differences between the existing literature and the hybrid method values: the food life cycle of goods modeled (hypotheses and calculation decisions made when conducting an LCA) and the characterization method itself. To explore these possibilities, an in-depth analysis was led on the ruminant meats category. A comparison of hybrid-method estimates against values found in the literature showed that differences were exclusively due to the GHGE impact of the beef meat. The median value of one kilogram of beef was 15.89 CO_2_eq./kg using the hybrid method and 27.56 CO_2_eq./kg using the existing literature data.

As the characterization method employed was the same in the different surveys, it is not the source of variation between literature values and estimate values. Although the published papers tended to be hazy on their calculation hypotheses, it did emerge that a key difference in terms of hypothesis was the country of production considered in each publication: in theory, country of production was never the same, but in reality, values employed to model livestock impact were often the same and thus not representative of the geographical situation of the system assessed. In fact, the median literature value was calculated on the basis of five GHGE values sourced from four publications: Audsley *et al*. [20], Carlsson-Kanyama *et al*. [41], Hoolohan *et al*. [39],and Roy *et al*. [42]. Audsley *et al*. [20] gave two values for the impact of 1 kg of beef consumed in the UK: 12 CO_2_eq/kg when production country was the UK and 32 kg CO_2_eq/kg when production country was Brazil. These data were already given (for the same production countries) by Williams *et al*. [43]. The value calculated for a specific Brazilian beef (i.e. 32 kg CO_2_eq./kg) not representative of Brazilian livestock as a whole was then re-employed in *Hoolohan et al*. [39] (for animal production and consumption in Japan) and Roy *et al*. [42] (for animal production in Brazil and consumption in the UK). These relationships between these different data explain their proximity without giving certainty as to their representativeness. For the impact value given by Carlsson-Kanyama *et al*. [39] (30 kg CO2e/kg), it was impossible to clearly establish the conditions and assumptions associated with the calculations performed. The problem arose with the value given by Williams *et al*. [43] for UK beef (i.e. 12 kg CO_2_e/kg), for which the hypotheses considered in this LCA study remained unclear.

The ruminant meat case perfectly illustrates the message of this article. On one hand, it would be inappropriate to use the GHGE of beef from Brazilian farms to represent the impact of French consumption when the French market is made up of 80% domestic production. On the other hand, without more information, how can we be expected to define the real values of French beef basing it on Audsley’s values which were given [20] for beef produced and consumed in the UK (12 kg CO2e/kg) or on Carlsson-Kanyama’s [41] values for beef produced and consumed in Sweden (30 kg CO2e/kg)? Ogino *et al* [44] had already signaled strong variability in GHGE values associated with beef, and Dollé *et al* [45] reached the same conclusion in an review of the literature referencing 21 publications giving GHGE impacts per kg of live beef, where values found ranged from 5 up to 25 kg CO2eq/kg of meat. The GHGE impact was shown to be highly dependent on breeding conditions: intensive/extensive, nature and geographic origins of feed supplies, animal species, duration of fattening periods, how carbon storage of the meadow is taken into account, and which impact allocation system is associated with the various co-products, i.e. calves, milk, meat, offal. Allocation choices are a big factor in explaining differences in GHGE values between the literature database and hybrid method database. Indeed, the system modeled with the hybrid method accounted for these co products which were found to drive a share of the GHGE impact of the carcass. This allocation scheme was defined in accordance with French market production and consumption including types of packaging, and can consequently be justified and/or modified as and when this market changes. Dollé *et al*’s [45] results were not integrated in our study as they only concern the living animals, but they complete our analysis. However, the variability exposed in Dollé et *al*’s [45] research mirrors the variability highlighted herein this paper. Taken together, these results illustrate the large variability in values employed to estimate the GHGE value of the beef in a French diet if no effort is engaged to standardize this value.

The value estimated here using the hybrid method (15.89 CO_2_e/kg) was consistent with the value given by Audsley *et al*. [20] for beef produced and consumed in the UK (12 kg CO2e/kg). With the hybrid method, the livestock impact of the European beef consumed in France was estimated on the basis of EcoInvent data. Reference farms were Swiss which is likely more appropriate than Brazilian data for reporting a French farming situation. Note too that the hybrid method framework is itself a good means to standardize the protocol used to estimate food impact. Indeed, having any such protocol is valuable in itself as it offers the opportunity to control the influence of each factor affecting variation in impact. It is essential to have this type of framework to effectively measure the influence of a change in breeding practices, beef origin, or types and power sources on the ultimate GHGE impact.

### Strengths and limitations of the study and the hybrid method used

Although food composition tables used in nutritional epidemiology may not be perfectly accurate, they do offer a frame of reference in which to assess nutrient intakes and look for solutions enabling continuous improvement of food consumption at country level. The environmental database built with the hybrid method presented here allowed us to meet these same needs on the environmental level.

Using standardized instead of literature-retrieved GHGE databases may lead to different results on sustainable diets. To our knowledge, only three epidemiology studies [46–48] did use standardized country-specific environmental data for food, and it is salient that their conclusions were somewhat contradictory with conclusions reached in studies using literature-retrieved GHGE databases: literature-based studies generally found a positive correlation between “healthy eating” and “environmental-friendly eating” [12, 13],[14–19] whereas studies based on standardized country-specific data did not systematically find this kind of relationship [46–48].

Another advantage of the hybrid method is that, like the classic LCA, it can also serve to assess impacts other than carbon emissions, such as eutrophication and air acidification impacts, as previously shown in the French case [49]. Given the importance and nature of the environmental impacts of the upstream life stages, there is a real interest in being able to run environmental evaluations that can simultaneously integrate these different indicators [22]. Environmental impact values obtained with the hybrid method were recently used to assess the GHGE impact of food consumption in France [46, 47]. The results showed that the hybrid method was well adapted for assessing the influence of food choices on the environmental impact of existing diets in a given country. It did this by estimating the environmental impact of self-selected diets and the relative contribution of different food categories to this impact [46]. It also served to study relationships between key aspects of diet sustainability, such as nutritional adequacy, affordability and environmental friendliness, in order to identify the more sustainable diets [47] and foods [49].

The present study does carry limitations. A first limitation is the selection of GHGE as sole indicator of the environmental impact of foods and beverages. Food production has many other environmental impacts, chiefly in terms of water consumption and pollution and loss of biodiversity, and the relevant French standard BPX 30-323-0 recommends using indicators of these impacts [35]. Nonetheless, although the hybrid method can handle the assessment of several indicators, including water footprint, few publications have actually given estimates for these indicators, and mostly using non-consistent methodologies. We were therefore unable to compare literature values against hybrid-method estimates on these indicators, as we did with GHGE.

A second limitation of this study, which leads out from this first limitation, is the literature review process conducted to extract food and beverage GHGE estimates from the literature. We may have missed some unpublished data or been unaware of relevant grey literature. Nevertheless, after carefully screening through the references retrieved from the database searches, we further included 18 references that were either already known to use or cited by the retrieved references. The objective of our literature search was more to show the variability in published GHGE estimates than to obtain true and absolute GHGE estimates for particular food groups. The brunt of the literature search was conducted by public health nutritionists rather than LCA experts. The risk of missing important references also highlights the difficulty for the wider scientific community to access and interpret LCA studies.

A third limitation concerns the importance of the upstream phases in environmental impacts and the diverse instantiation of the values of these impacts depending on cultivation and breeding patterns. The next task to pursue could be to standardize the food categories used in this study in order to differentiate products according to upstream crop or livestock practices (organic farming, rational and intensive, intensive/extensive farming). Another limitation stems from the nutritional categorization of food items used by the authors at the start of the study. Different meats, for example, were all pooled into one category. Given the variability of GHGE impacts, meats were then separated into two categories: white and red meats. Finally, we differentiated ruminant from non-ruminant categories according to their GHGE impacts. Furthermore, the categories used in the present study included both unprocessed and processed foods, sterilized foods, and cold foods (frozen foods for example), all of which involve different methods with very different impacts on GHGE levels. Work to redefine the food categories is therefore needed for more accurate environmental impact values.

### Potential applications and further developments of the hybrid method

The evaluation process needs to be computerized to reduce the cost and duration of environmental assessments. An automated process can be expected to quickly increase the quantities of environmental data on food products and thus complete the databases available for nutritional studies. This additional data should also unlock differentiated data on factors identified as most influential–as seen for animal origin and the allocation of the impact to co-products in the case of beef. A “big data trend” would likely play a major role in driving the creation of an extended database by multiplying the data flows available for it. Data expansion should also reach into the usage/consumption step of the product life cycle. However these changes in themselves are not enough.

The reduction of processing time is not the only reason to use a highly structured environmental assessment process like the hybrid method. As shown by our analysis of meat GHGE impacts, there are relatively many criteria influencing the final impact due to the multiplicity of assumptions and conditions that are associated with a LCA, which means a lot of information has to be gathered to clarify the model evaluated. Developing the cognitive process needed to sift through this data is not in line with the objectives, and time constraints, of nutritional and public health studies. The only way to decrease the complexity involved would be to reduce the number of alternatives and assumptions that have to be taken into account for each study, as proposed in Le Pochat *et al*.[50] and Andriankaja *et al*[51]. There is need for modelling and calculation procedures that reduce the non-mastered variability of environmental studies.

In this perspective, the hybrid model offers real framework for environmental assessments on food products, in line with European Commission recommendations on how to communicate product life cycle environmental performances [33, 52, 53]. As the role of the Product Category Rules (PCRs) is to establish the conditions and assumptions to apply when conducting a given LCA the estimated impacts do not have to be exact, or true, just probable and repeatable. The issue–in the context of epidemiology surveys–is more about measuring the impacts of changes in eating habits or agricultural practices than to getting a “true” product impact value.

European recommendations [33] stipulate that the environmental assessment has to be multi-criteria, which is possible with the hybrid method. This French study highlights the importance of creating product databases following national standards using reliable sources and representative data, as has already been used for food nutritional value databases, in order to improve the quantitative assessment of the environmental impacts of foods and diets. The methodology presented here could be applied in other countries or regions where there is appropriate available environmental impact data and macro-economic data. Use of the hybrid method could therefore help build these needed national standard databases.

## Conclusion

The hybrid input-output/LCA standardized method presented in this study served to build a database containing accurate GHGE values for foods representative of the French national market. Using this environmental database rather than GHGE values retrieved from the heterogeneous literature conducted under different LCA modeling hypotheses will likely not only simplify but also improve the assessment of the environmental impacts of diets. From a public health perspective, hybrid method could help strengthen the study of diet sustainability and identify more sustainable diets.

## Supporting Information

S1 Fig Existing literature and hybrid method GHGE calculations distribution across food categories. (DOCX)

S1 List References retrieved from the existing literature search and grey literature. (DOCX)

S1 Table Median GHGE (in g CO_2_eq/kg) estimates from existing literature and from the hybrid method, by food category. (DOCX)

S2 Table Illustration of GHGE results and data used with the hybrid method, applied to the ruminant meats category (Functional Unit of 1kg). (DOCX)

S3 Table Data retrieved from scientific literature for the ruminant meats category (Functional Unit of 1kg). (DOCX)
