# Supplementary material for: How to Build a Standardized Country-Specific Environmental Food Database for Nutritional Epidemiology Studies
Source: PLoS One. 2016 Apr 7;11(4):e0150617. doi: 10.1371/journal.pone.0150617 (PMC4824438; doi:10.1371/journal.pone.0150617)
Supplement: S1 Fig — (DOCX) [file pone.0150617.s001.docx]

**Fig S1. Existing literature and hybrid method GHGE calculations distribution across food categories.** The boxplots represent the interquartile range and extreme values by food category. The median for each food category is indicated by the horizontal line within the boxes. *Median estimates extracted from the existing literature or the hybrid method calculations are significantly different (p sign-test <0.05).


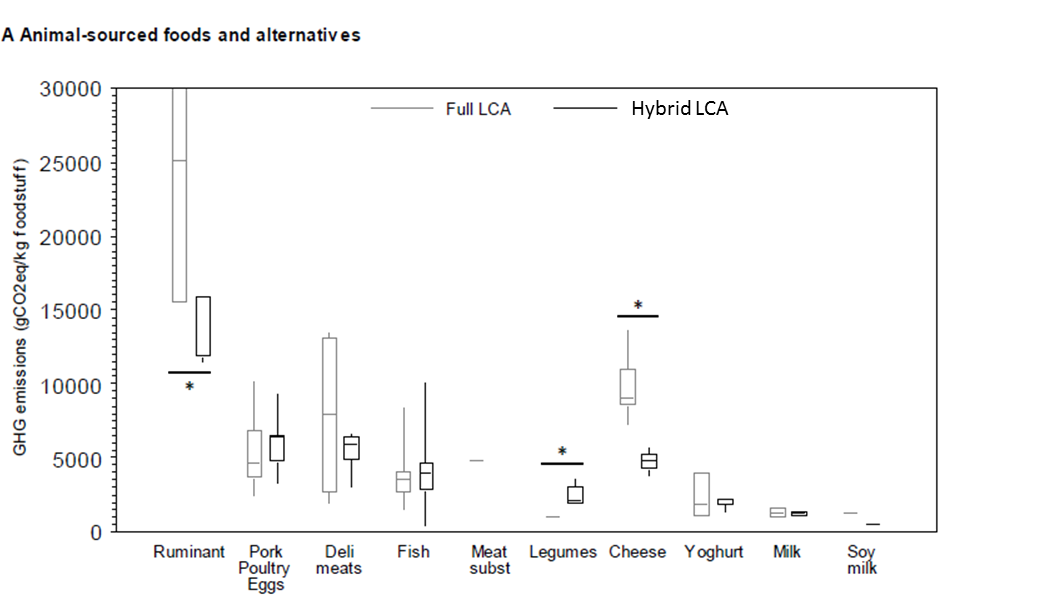


Existing literature

Hybrid

LCA


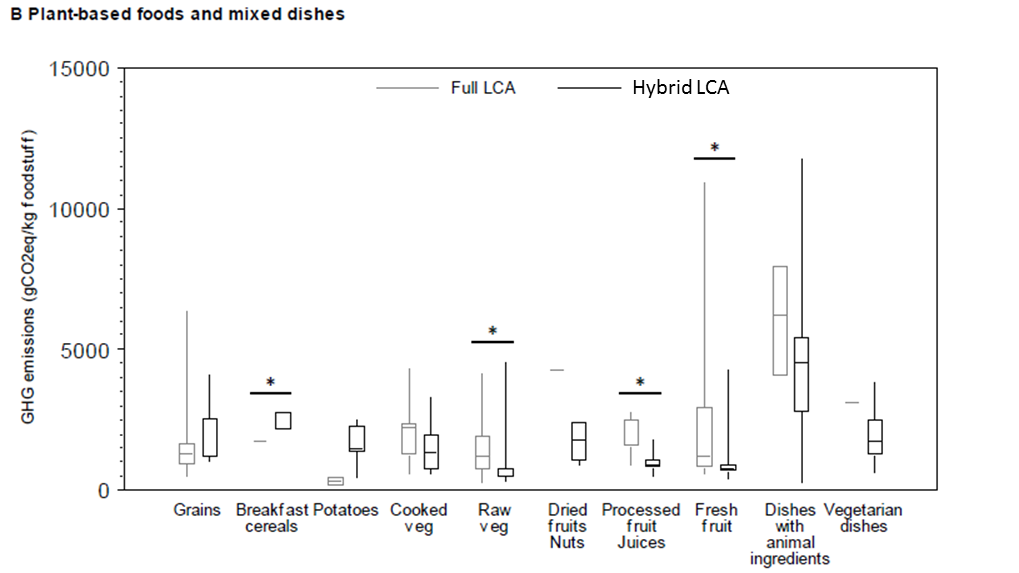


Existing literature

Hybrid

LCA


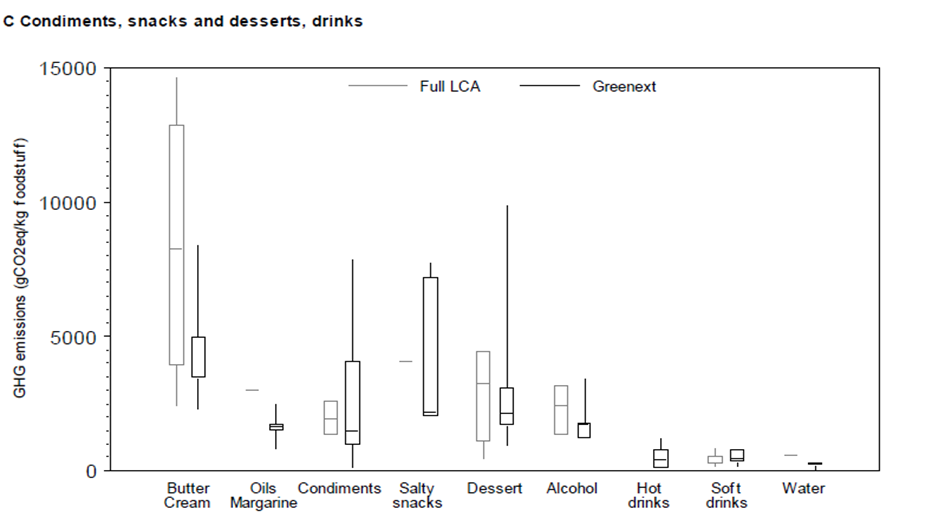


Existing literature

Hybrid

LCA
