# Supplementary material for: How to Build a Standardized Country-Specific Environmental Food Database for Nutritional Epidemiology Studies
Source: PLoS One. 2016 Apr 7;11(4):e0150617. doi: 10.1371/journal.pone.0150617 (PMC4824438; doi:10.1371/journal.pone.0150617)
Supplement: S1 List — (DOCX) [file pone.0150617.s002.docx]

**S1 List References retrieved from the existing literature search and grey literature**

1. Amienyo D, Gujba H, Stichnothe H, Azapagic A. Life cycle environmental impacts of carbonated soft drinks. *Int J Life Cycle Assess*. 2012 Jul 3;**18**(1):77–92.

2. Audsley E, Brander M, Chatterton J, Murphy-Bokern D, Webster C, Williams A. How low can we go? An assessment of greenhouse gas emissions from the UK food system and the scope for reduction by 2050. Godalming, UK: FCRN-WWF-UK; 2010.

3. Basset-Mens C, Kelliher FM, Ledgard S, Cox N. Uncertainty of global warming potential for milk production on a New Zealand farm and implications for decision making. *Int J Life Cycle Assess*. 2009 Sep 1;**14**(7):630–638.

4. Beauchemin KA, Janzen HH, Little SM, McAllister TA, McGinn SM. Mitigation of greenhouse gas emissions from beef production in western Canada – Evaluation using farm-based life cycle assessment. *Anim Feed Sci Technol*. 2011 Jun;**166-167**:663–677.

5. Bengtsson J, Seddon J. Cradle to retailer or quick service restaurant gate life cycle assessment of chicken products in Australia. *J Clean Prod*. 2013 Feb;**41**:291–300.

6. Blonk H, Kool A, Luske B, Waarf S de. Environmental effects of protein-rich food products in the Netherlands. Consequences of animal protein substitutes. Gouda, NL; 2008.

7. Bonesmo H, Beauchemin KA, Harstad OM, Skjelvåg AO. Greenhouse gas emission intensities of grass silage based dairy and beef production: A systems analysis of Norwegian farms. *Livest Sci*. 2013 Apr;**152**(2-3):239–252.

8. Brito de Figueirêdo MC, Kroeze C, Potting J, da Silva Barros V, Sousa de Aragao FA, Sonsol Gondim R et al. The carbon footprint of exported Brazilian yellow melon. *J Clean Prod*. 2013 May;**47**:404–414.

9. Brodt S, Kramer KJ, Kendall A, Feenstra G. Comparing environmental impacts of regional and national-scale food supply chains: A case study of processed tomatoes. *Food Policy*. 2013 Oct;**42**:106–114.

10. Carlsson-Kanyama A, González AD. Potential contributions of food consumption patterns to climate change. *Am J Clin Nutr*. 2009 May;**89**(5):1704S–1709S.

11. Cederberg C, Hedenus F, Wirsenius S, Sonesson U. Trends in greenhouse gas emissions from consumption and production of animal food products - implications for long-term climate targets. *Animal*. 2013 Feb;**7**(2):330–40.

12. Cellura M, Ardente F, Longo S. From the LCA of food products to the environmental assessment of protected crops districts: a case-study in the south of Italy. *J Environ Manage*. 2012 Jan;**93**(1):194–208.

13. Vries M de, Boer IJM de. Comparing environmental impacts for livestock products: A review of life cycle assessments. *Livest Sci*. 2010 Mar;**128**(1-3):1–11.

14. Desjardins R, Worth D, Vergé X, Maxime D, Dyer J, Cerkowniak D. Carbon footprint of beef cattle. *Sustainability*. 2012 Dec 3;**4**(12):3279–3301.

15. Dick M, Abreu da Silva M, Dewes H. Life cycle assessment of beef cattle production in two typical grassland systems of southern Brazil. *J Clean Prod*. 2014 Feb;

16. Dollé J, Agabriel J, Peyraud J, Faverdin P, Manneville V, Raison C, et al. Greenhouse gases in cattle breeding: evaluation and mitigation strategies. *INRA Prod Anim*. 2011;**24**(5):415–432.

17. Dollé J, Manneville V, Gac A, Charpiot A. Emissions de gaz à effet de serre et consommations d’énergie des viandes bovines et ovines françaises : revue bibliographique et évaluations sur l'amont agricole. Paris, France; 2011.

18. Dwivedi P, Spreen T, Goodrich-Schneider R. Global warming impact of Florida’s Not-From-Concentrate (NFC) orange juice. *Agric Syst*. 2012 Apr;**108**:104–111.

19. Ecointesys. Revue bibliographique des études “Analyse de Cycle de Vie des produits agricoles”. Summary report. Angers, France; 2008.

20. Flysjö A. Potential for improving the carbon footprint of butter and blend products. *J Dairy Sci*. 2011 Dec;**94**(12):5833–41.

21. Flysjö A, Thrane M, Hermansen JE. Method to assess the carbon footprint at product level in the dairy industry. *Int Dairy J*. 2014 Jan;**34**(1):86–92.

22. Foster C, Grenn K, Bleda M, Dewick P, Evans B, Flynn A, et al. Environmental Impacts of Food Production and Consumption - Final Report to the Department for Environment, Food and Rural Affairs. London; 2006.

23. Gerber P, Steinfeld H, Henderson B, Mottet A, Opio C, Dijkman J, et al. Tackling climate change through livestock - A global assessment of emissions and mitigation opportunities. Rome, Italy: Food and Agriculture Organization; 2013.

24. González AD, Frostell B, Carlsson-Kanyama A. Protein efficiency per unit energy and per unit greenhouse gas emissions: Potential contribution of diet choices to climate change mitigation. *Food Policy*. 2011 Oct;**36**(5):562–570.

25. González-García S, Castanheira ÉG, Dias AC, Arroja L. Environmental performance of a Portuguese mature cheese-making dairy mill. *J Clean Prod*. 2013 Feb;**41**:65–73.

26. González-García S, Castanheira ÉG, Dias AC, Arroja L. Environmental life cycle assessment of a dairy product: the yoghurt. *Int J Life Cycle Assess*. 2012 Oct 25;

27. Guignard C, Verones F, Loerincik Y, Jolliet O. Environmental/ecological impact of the dairy sector: Literature review on dairy products for an inventory of key issues. List of environmental initiatives and influences on the dairy sector. *Bull Int Dairy Fed*. 2009;**436**.

28. Hagemann M, Ndambi A, Hemme T, Latacz-Lohmann U. Contribution of milk production to global greenhouse gas emissions. An estimation based on typical farms. *Environ Sci Pollut Res Int*. 2012 Feb;**19**(2):390–402.

29. Head M, Sevenster M, Croezen H. Life Cycle Impacts of Protein-rich Foods for Superwijzer. Delft, NL; 2011.

30. Hoolohan C, Berners-Lee M, McKinstry-West J, Hewitt CN. Mitigating the greenhouse gas emissions embodied in food through realistic consumer choices. *Energy Policy*. Elsevier; 2013 Oct;1–10.

31. Hospido A, Milà i Canals L, McLaren S, Truninger M, Edwards-Jones G, Clift R. The role of seasonality in lettuce consumption: a case study of environmental and social aspects. *Int J Life Cycle Assess*. 2009 May 29;**14**(5):381–391.

32. Ingwersen WW. Life cycle assessment of fresh pineapple from Costa Rica. *J Clean Prod* . 2012 Nov;**35**:152–163.

33. Iriarte A, Almeida MG, Villalobos P. Carbon footprint of premium quality export bananas: case study in Ecuador, the world’s largest exporter. *Sci Total Environ*. 2014 Feb 15;**472**:1082–8.

34. Iribarren D, Vázquez-Rowe I, Hospido A, Moreira MT, Feijoo G. Estimation of the carbon footprint of the Galician fishing activity (NW Spain). *Sci Total Environ*. 2010 Oct 15;**408**(22):5284–94.

35. Kendall A, Yuan J, Brodt SB. Carbon footprint and air emissions inventories for US honey production: case studies. *Int J Life Cycle Assess*. 2012 Sep 1;**18**(2):392–400.

36. Kim D, Thoma G, Nutter D, Milani F, Ulrich R, Norris G. Life cycle assessment of cheese and whey production in the USA. *Int J Life Cycle Assess*. 2013 Feb 19;**18**(5):1019–1035.

37. Lesschen JP, Berg M van den, Westhoek HJ, Witzke HP, Oenema O. Greenhouse gas emission profiles of European livestock sectors. *Anim Feed Sci Technol*. 2011 Jun;**166-167**:16–28.

38. McGeough EJ, Little SM, Janzen HH, McAllister TA, McGinn SM, Beauchemin KA. Life-cycle assessment of greenhouse gas emissions from dairy production in Eastern Canada: a case study. *J Dairy Sci*. 2012 Sep;**95**(9):5164–75.

39. Meier T, Christen O. Gender as a factor in an environmental assessment of the consumption of animal and plant-based foods in Germany. *Int J Life Cycle Assess*. 2012 Feb 21;**17**(5):550–564.

40. Modernel P, Astigarraga L, Picasso V. Global versus local environmental impacts of grazing and confined beef production systems. *Environ Res Lett*. 2013 Sep 1;**8**(3):035052.

41. Nijdam D, Rood T, Westhoek H. The price of protein: Review of land use and carbon footprints from life cycle assessments of animal food products and their substitutes. *Food Policy*. 2012 Dec;**37**(6):760–770.

42. Nilsson K, Flysjö A, Davis J, Sim S, Unger N, Bell S. Comparative life cycle assessment of margarine and butter consumed in the UK, Germany and France. *Int J Life Cycle Assess*. 2010 Aug 26;**15**(9):916–926.

43. Noponen MR, Edwards-Jones G, Haggar JP, Soto G, Attarzadeh N, Healey JR. Greenhouse gas emissions in coffee grown with differing input levels under conventional and organic management. *Agric Ecosyst Environ*. 2012 Apr;**151**:6–15.

44. Oonincx DGAB, de Boer IJM. Environmental impact of the production of mealworms as a protein source for humans – A life cycle assessment. *PLoS One*. 2012 Dec 19;**7**(12):e51145.

45. Page G, Ridoutt B, Bellotti B. Carbon and water footprint tradeoffs in fresh tomato production. *J Clean Prod*. 2012 Sep;**32**:219–226.

46. Pattara C, Raggi A, Cichelli A. Life cycle assessment and carbon footprint in the wine supply-chain. *Environ Manage*. 2012 Jun;**49**(6):1247–58.

47. Pelletier N, Pirog R, Rasmussen R. Comparative life cycle environmental impacts of three beef production strategies in the Upper Midwestern United States. *Agric Syst*. 2010 Jul;**103**(6):380–389.

48. Pelletier N, Tyedmers P, Sonesson U, Scholz A, Ziegler F, Flysjo A et al. Not all salmon are created equal: life cycle assessment (LCA) of global salmon farming systems. *Environ Sci Technol*. 2009 Dec 1;**43**(23):8730–6.

49. Reckmann K, Traulsen I, Krieter J. Life Cycle Assessment of pork production: A data inventory for the case of Germany. *Livest Sci*. 2013 Nov;**157**(2-3):586–596.

50. Ridoutt BG, Sanguansri P, Harper GS. Comparing carbon and water footprints for beef cattle production in Southern Australia. *Sustainability*. 2011 Dec 13;**3**(12):2443–2455.

51. Ripoll-Bosch R, de Boer IJM, Bernués A, Vellinga TV. Accounting for multi-functionality of sheep farming in the carbon footprint of lamb: A comparison of three contrasting Mediterranean systems. *Agric Syst*. 2013 Mar;**116**:60–68.

52. Röös E, Karlsson H. Effect of eating seasonal on the carbon footprint of Swedish vegetable consumption. *J Clean Prod*. 2013 Nov;**59**:63–72.

53. Röös E, Sundberg C, Hansson P-A. Uncertainties in the carbon footprint of refined wheat products: a case study on Swedish pasta. *Int J Life Cycle Assess*. 2011 Mar 23;**16**(4):338–350.

54. Röös E, Sundberg C, Hansson P-A. Uncertainties in the carbon footprint of food products: a case study on table potatoes. *Int J Life Cycle Assess*. 2010 Mar 19;**15**(5):478–488.

55. Roy P, Orikasa T, Thammawong M, Nakamura N, Xu Q, Shiina T. Life cycle of meats: an opportunity to abate the greenhouse gas emission from meat industry in Japan. *J Environ Manage*. 2012 Jan;**93**(1):218–24.

56. Rugani B, Vázquez-Rowe I, Benedetto G, Benetto E. A comprehensive review of carbon footprint analysis as an extended environmental indicator in the wine sector. *J Clea Prod*. 2013 Sep;**54**:61–77.

57. Saunders C, Barber A. Carbon footprints, life cycle analysis, food miles: global trade trends and market issues. *Polit Sci*. 2008 Jun 1;**60**(1):73–88.

58. Svanes E, Aronsson AKS. Carbon footprint of a Cavendish banana supply chain. *Int J Life Cycle Assess*. 2013 Jun 6;**18**(8):1450–1464.

59. Tan MQB, Tan RBH, Khoo HH. Prospects of carbon labelling – a life cycle point of view. *J Clean Prod*. 2012 Oct;

60. Taylor RC, Omed H, Edwards-Jones G. The greenhouse emissions footprint of free-range eggs. *Poult Sci*. 2014 Jan;**93**(1):231–7.

61. Teixeira R, Himeno A, Gustavus L. Carbon footprint of Breton pâté production: a case study. *Integr Environ Assess Manag*. 2013 Oct;**9**(4):645–51.

62. Tuomisto HL, Mattos MJT de. Environmental impacts of cultured meat production. *Environ Sci Technol*. 2011 Jul 15;**45**(14):6117–23.

63. Vázquez-Rowe I, Moreira MT, Feijoo G. Carbon footprint analysis of goose barnacle (Pollicipes pollicipes) collection on the Galician coast (NW Spain). *Fish Res*. 2013 Jun;**143**:191–200.

64. Vázquez-Rowe I, Rugani B, Benetto E. Tapping carbon footprint variations in the European wine sector. *J Clean Prod*. 2013 Mar;**43**:146–155.

65. Vázquez-Rowe I, Villanueva-Rey P, Mallo J, la Cerda JJ De, Moreira MT, Feijoo G. Carbon footprint of a multi-ingredient seafood product from a business-to-business perspective. *J Clean Prod*. 2013 Apr;**44**:200–210.

66. Wallén A, Brandt N, Wennersten R. Does the Swedish consumer’s choice of food influence greenhouse gas emissions? *Environ Sci Policy*. 2004 Dec;**7**(6):525–535.

67. Webb J, Williams AG, Hope E, Evans D, Moorhouse E. Do foods imported into the UK have a greater environmental impact than the same foods produced within the UK? *Int J Life Cycle Assess*. 2013 Apr 24;**18**(7):1325–1343.

68. Williams AG, Audsley E, Sandars DL. Determining the environmental burdens and resource use in the production of agricultural and horticultural commodities. Defra project report IS0205. Bedford, UK; 2006.

69. Williams AG, Audsley E, Sandars DL. Environmental burdens of producing bread wheat, oilseed rape and potatoes in England and Wales using simulation and system modelling. *Int J Life Cycle Assess*. 2010 Jul 3;**15**(8):855–868.

70. Ziegler F, Winther U, Hognes ES, Emanuelsson A, Sund V, Ellingsen H. The carbon footprint of Norwegian seafood products on the global seafood market. *J Ind Ecol*. 2013 Feb 23;**17**(1):103–116.
