# Supplementary material for: How to Build a Standardized Country-Specific Environmental Food Database for Nutritional Epidemiology Studies
Source: PLoS One. 2016 Apr 7;11(4):e0150617. doi: 10.1371/journal.pone.0150617 (PMC4824438; doi:10.1371/journal.pone.0150617)
Supplement: S1 Table — (DOCX) [file pone.0150617.s003.docx]

**S1 Table Median GHGE (in g CO_2_eq/kg) estimates from existing literature and from the hybrid method, by food category**

|  | Hybrid Method | |  | Literature | |  |
| --- | --- | --- | --- | --- | --- | --- |
|  | N | Median |  | N | Median | p* |
| Ruminant | 10 | 15892 |  | 4 | 27565 | **0.039** |
| Pork, poultry, eggs | 9 | 6341 |  | 10 | 4600 | 0.369 |
| Deli 1meats | 12 | 5922 |  | 4 | 7900 | 0.903 |
| Fish | 26 | 3867 |  | 6 | 3575 | 0.530 |
| Meat substitutes |  |  |  | 1 | 4810 |  |
| Legumes | 7 | 2026 |  | 1 | 920 | 0.124 |
| Cheese | 28 | 4754 |  | 5 | 9000 | **<0.001** |
| Milk | 4 | 1252 |  | 3 | 1180 | 0.724 |
| Yoghurt | 10 | 1810 |  | 3 | 1776 | 0.494 |
| Soy milk | 1 | 521 |  | 1 | 1260 |  |
| Grains | 22 | 1203 |  | 9 | 1300 | 0.458 |
| Potatoes | 5 | 1469 |  | 5 | 305 | **0.016** |
| Breakfast cereals | 5 | 2749 |  | 1 | 1730 | 0.114 |
| Cooked vegetables | 34 | 1314 |  | 6 | 2215 | 0.240 |
| Raw vegetables | 14 | 743 |  | 20 | 1189 | **0.014** |
| Dried fruits and nuts | 8 | 1751 |  | 1 | 4260 | 0.121 |
| Processed fruit and juices | 12 | 917 |  | 5 | 1610 | **0.044** |
| Fresh fruits | 24 | 781 |  | 11 | 1210 | **0.016** |
| Mixed dishes, with animal ingredients | 35 | 4509 |  | 3 | 6200 | 0.167 |
| Vegetarian mixed dishes | 9 | 1720 |  | 1 | 3110 | 0.222 |
| Butter, cream | 5 | 3475 |  | 4 | 8290 | 0.219 |
| Vegetable oils, Margarine | 10 | 1672 |  | 1 | 3000 | 0.112 |
| Condiments | 16 | 1483 |  | 2 | 1972.5 | 0.673 |
| Salty snacks | 6 | 2164 |  | 1 | 4090 | 0.604 |
| Dessert | 55 | 2130 |  | 9 | 3270 | 0.854 |
| Alcohol | 10 | 1704 |  | 3 | 2410 | 0.304 |
| Hot drinks | 8 | 390 |  | 0 |  |  |
| Soft drinks | 6 | 447 |  | 5 | 312 | 0.584 |
| Water | 11 | 250 |  | 1 | 580 | 0.073 |

*p values for Kruskal-Wallis tests.
