# Supplementary material for: How to Build a Standardized Country-Specific Environmental Food Database for Nutritional Epidemiology Studies
Source: PLoS One. 2016 Apr 7;11(4):e0150617. doi: 10.1371/journal.pone.0150617 (PMC4824438; doi:10.1371/journal.pone.0150617)
Supplement: S2 Table — (DOCX) [file pone.0150617.s004.docx]

**S2 Table Illustration of GHGE results and data used with the hybrid method, applied to the ruminant meats category (Functional Unit of 1kg).**

| **Product** | **Results Kg CO_2_eq /kg** | **Characterization method** | **LCI inventories database** | **System boundaries** | **Allocation rules** | **Production Country** | **Consumption country** | **Additional Information** |
| --- | --- | --- | --- | --- | --- | --- | --- | --- |
| Grilled lamb chop | 11,83 | IPCC 2007 GWP 100a, November 2009, V1.02 | ESU database: lamb, IP, at farm/CH U | Phase of cattle farm to usage phase | Mass | Switzerland | France | Ingredient inventories LCA come from ESU database |
| Cooked beef | 15,891 | IPCC 2007 GWP 100a, November 2009, V1.02 | ESU database: cattle, IP, at farm/CH U | Phase of cattle farm to usage phase | Mass | Switzerland | France | Ingredient inventories LCA come from ESU database |
| Cooked veal cutlet | 11,45 | IPCC 2007 GWP 100a, November 2009, V1.02 | ESU database: calf, IP, at farm/CH U | Phase of cattle farm to usage phase | Mass | Switzerland | France | Ingredient inventories LCA come from ESU database |
| Cooked veal liver | 11,45 | IPCC 2007 GWP 100a, November 2009, V1.02 | ESU database: calf, IP, at farm/CH U | Phase of cattle farm to usage phase | Mass | Switzerland | France | Ingredient inventories LCA come from ESU database |
| Grilled beef steak | 15,89 | IPCC 2007 GWP 100a, November 2009, V1.02 | ESU database: cattle, IP, at farm/CH U | Phase of cattle farm to usage phase | Mass | Switzerland | France | Ingredient inventories LCA come from ESU database |
| Cooked beef burger 15% fat | 15,89 | IPCC 2007 GWP 100a, November 2009, V1.02 | ESU database: cattle, IP, at farm/CH U | Phase of cattle farm to usage phase | Mass | Switzerland | France | Ingredient inventories LCA come from ESU database |
| Cooked beef burger 10% fat | 15,89 | IPCC 2007 GWP 100a, November 2009, V1.02 | ESU database: cattle, IP, at farm/CH U | Phase of cattle farm to usage phase | Mass | Switzerland | France | Ingredient inventories LCA come from ESU database |
| Cooked beef burger 5% fat | 15,89 | IPCC 2007 GWP 100a, November 2009, V1.02 | ESU database: cattle, IP, at farm/CH U | Phase of cattle farm to usage phase | Mass | Switzerland | France | Ingredient inventories LCA come from ESU database |
| Roasted leg of lamb | 11,83 | IPCC 2007 GWP 100a, November 2009, V1.02 | ESU database: lamb, IP, at farm/CH U | Phase of cattle farm to usage phase | Mass | Switzerland | France | Ingredient inventories LCA come from ESU database |
