# Supplementary material for: How to Build a Standardized Country-Specific Environmental Food Database for Nutritional Epidemiology Studies
Source: PLoS One. 2016 Apr 7;11(4):e0150617. doi: 10.1371/journal.pone.0150617 (PMC4824438; doi:10.1371/journal.pone.0150617)
Supplement: S3 Table — (DOCX) [file pone.0150617.s005.docx]

**S3 Table Data retrieved from scientific literature for the ruminant meats category (Functional Unit of 1kg).**

| **Source** | **Results Kg CO_2_eq /kg** | **Characterization method** | **LCI inventories database** | **System boundaries** | **Production Country** | **Consumption country** | **Additional Information** |
| --- | --- | --- | --- | --- | --- | --- | --- |
| Audsley (2010) | 32 | IPCC 2006, GWP 100y | Primary data+previous data+ EcoInvent | Farm to regional distribution center | Brazil | UK | Averaged data & including land use |
| Audsley (2010) | 12 | IPCC 2006, GWP 100y | Primary data+previous data+ EcoInvent | Farm to regional distribution center | Brazil | UK | Averaged data & allocation dairy cow |
| Carlsson-Kanyama & al (2009) | 30 | IPCC not defined, GWP 100y | Swedish primary data, centered on energy consumption | Farm to use phase, cooked beef | Sweden | Sweden | Losses taking into account at all stages |
| Hoolohan (2013) | 25 | IPCC 2006, GWP 100y | Williams (2008) (farm step) & National data base packaging materials & transport. | Farm to regional distribution center | Brazil | UK | Detailed model of distribution and frozen storage |
| Roy  (2012) | 25 | IPCC 2006, GWP 100y | Williams (2008) (farm step) & National data base packaging materials & transport | Farm to use phase, | Japan | Japan | Only 40% of the living animal is considered meat |
